# Co-registration of Sequential Multidetector Computed Tomography Studies for the Evaluation of Surgical Instrumentation following Resection of Spinal Tumors

**DOI:** 10.1155/2011/676410

**Published:** 2011-07-18

**Authors:** J. Matthew Debnam, T. Linda Chi, Leena Ketonen, Yasser M. M. Mahfouz, Nandita Guha-Thakurta

**Affiliations:** ^1^Section of Neuroradiology, Department of Diagnostic Radiology, The University of Texas MD Anderson Cancer Center, 1400 Pressler, Unit 1482, Houston, TX 77030, USA; ^2^Department of Radiology, National Cancer Institute, Cairo University, Kasr El-Ainy street, Fom Elkhalig- Elsaieda Zainab, Cairo 11141, Egypt

## Abstract

Surgical resection of spinal tumors involves complex reconstructive procedures. The stability and integrity of the surgical construct are evaluated with multidetector computed tomography (MDCT). As coregistration, or fusion, of different imaging modalities, especially positron emission tomography/computed tomography (PET/CT), is common practice, we sought to determine if this technique could be applied to sequential, postoperative MDCT studies of the spine. Herein, we demonstrate that by utilizing the Hermes workstation, co-registration of MDCT spine studies can be performed. This technique allows sequential MDCT examinations of the post-operative spine to be viewed together as one study and may aid in evaluation of the position and integrity of the surgical construct over time. Further study and refinement of this technique will be necessary before clinical implementation.

## 1. Introduction

Surgical resection is often performed as part of the treatment for primary and metastatic spinal tumors. This includes tumor resection and the placement of complex metallic and bony constructs for stabilization and support. Infrequent complications related to spinal instrumentation, including those of pedicular screws and plating systems, have been reported in the literature [1–3]. Early radiological detection of construct failure enables surgical correction, if necessary, potentially avoiding complications which may lead to neurological compromise. Computed tomography (CT) has been used to evaluate surgical instrumentation, including the spine, and has been described for the assessment of surgical fusion [4–6] and subsequent trabeculation [6, 7] as well as associated complications [8]. 

Co-registration of various imaging modalities has been performed, including positron emission tomography (PET), CT, and magnetic resonance imaging (MRI) [9–11]. This technique can be performed on a Hermes workstation (Hermes Medical Solutions, Stockholm, Sweden) and allows images from two separate studies, such as a CT and PET, to be overlaid and viewed together as one study, that is, a PET/CT scan. A recent article has described using CT/CT co-registration for the evaluation of bone mineral density along screw trajectories [12]. 

Detection of subtle changes in hardware position is imperative and can be difficult. Therefore, we investigated to determine if co-registration could also be performed with sequential multidetector computed tomography (MDCT) examinations of the spine. Herein, we describe our initial experience using this co-registration technique for the MDCT evaluation of the surgical construct in patients after resection of spinal tumors and report on the first two cases where this technique was utilized to evaluate the postoperative spine. 

## 2. Technique

Sequential MDCT imaging was performed on a 16-slice multidetector CT scanner (GE LightSpeed, GE Medical Systems, Milwaukee, Wis), with the following parameters: 140 kV, 220 to 250 mA, and a 1.25 mm collimation. As our protocol utilizes a wide field of view (250–300 mm), images are cropped in the *x*-*y* plane and along the *z*-axis, to include only the surgical construct and adjacent bony structures. The cropped studies are then sent to and coregistered, or fused, automatically on a Hermes workstation using a program named Rapid Viewer (Hermes Medical Solutions, Stockholm, Sweden). This program allows for further manipulation of the coregistered images, that is, moving one of the images in the *x*-*y* plane, or *z*-axis, to ensure proper alignment between the two studies. Various color schemes and inverse patterns are available for viewing. 

## 3. Illustrative Cases

**Case 1**

**Figure 1:**
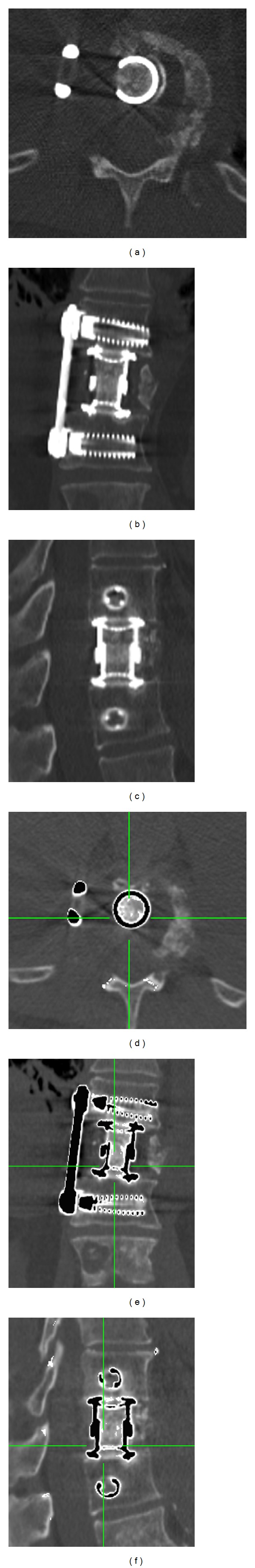
Case 1: 79-year-old man with metastatic leiomyosarcoma to the T11 vertebral body, s/p vertebrectomy, and reconstruction. (a, b, c). Axial, coronal, and sagittal MDCT. The position of the expandable titanium cage and anterior thoracic fusion plate is demonstrated. (d, e, f). Co-registration of sequential MDCT studies. The original study is in white color and the more recent study is in black color; a similar position of the surgical instrumentation between the two sequential studies is demonstrated. Green line denotes corresponding position of the other two orthogonal planes.

A 79-year-old man with metastatic leiomyosarcoma to the T11 vertebral body underwent tumor resection, including a T11 vertebrectomy. Reconstruction of the T11 level was performed with an expandable titanium cage, and the spine was stabilized anteriorly from T10–T12 using a thoracic plate. The construct was stable on followup imaging between the immediate postsurgical imaging and at followup 6 months later (Figure 1).

**Case 2**

**Figure 2:**
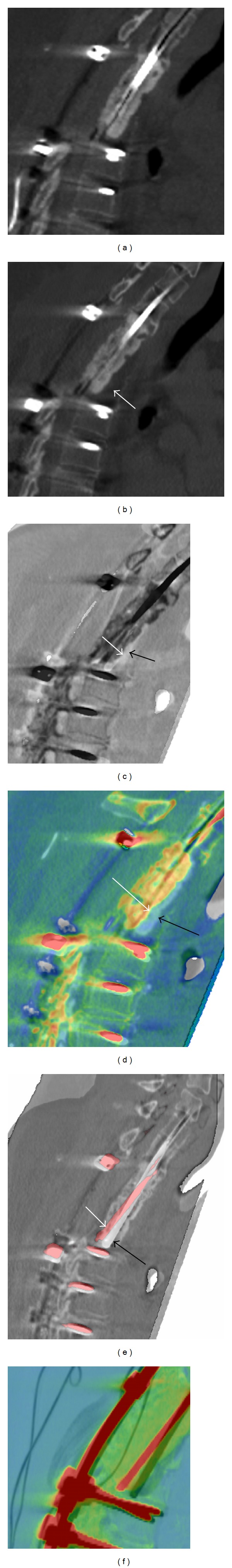
Case 2: 39-year-old man with renal cell carcinoma metastatic to the T2 through T4 vertebral bodies, s/p vertebrectomy with placement of Steinmann pin and methyl methacrylate graft. (a, b). Sequential sagittal noncontrast MDCT studies 3 months apart demonstrate subtle posterior migration of the Steinmann pin and methyl methacrylate graft. Arrow in (b) points to initial position of the graft. (c, d). Sagittal co-registered MDCT studies (windowed to view methyl methacrylate). Posterior migration of the methyl methacrylate graft; initial position (black arrow) and position on followup, (white arrow). (e) Sagittal coregistered MDCT studies (windowed to view instrumentation). Posterior migration of the Steinmann pin; initial position (black arrow) and position on followup (white arrow). (f). MIP (maximal intensity projection). Posterior migration of the Steinmann pin; original position: black color, followup: red color.

A 39-year-old man with renal cell carcinoma metastatic to the T2 through T4 vertebral bodies underwent surgical resection of the tumor. Vertebrectomy from T2 through T4 was performed, and the operative site was stabilized using a Steinmann pin, methyl methacrylate graft, and posterior instrumentation. On followup imaging, obtained 3 months after surgery, the inferior aspect of the Steinmann pin and the methyl methacrylate graft, which were positioned on the superior T5 endplate, had migrated posteriorly (Figure 2).

## 4. Discussion

We have demonstrated that co-registration of sequential MDCT studies of the spine is technically feasible and allows separate studies of the postoperative spine to be viewed together as one image. While instrumentation failure is rare, the results can be devastating for the patient, and prompt detection is mandatory. Radiologists and spine surgeons who interpret MDCT imaging of the postoperative spine are required to assess for subtle changes in position of the surgical construct over time. As technology advances, the ability to evaluate the metallic portion of the surgical construct has improved [13, 14]; however, the complexity of the studies and interpretation time has increased, as well. Co-registration allows radiologists and spine surgeons to directly view two different MDCT studies of the surgical construct as one image set and evaluate stability over time, or detect subtle changes in instrument and/or bony position. Different color schemes are available for visualization in three orthogonal planes, depending on the preference of the interpreting physician. A Maximal Intensity Projection (MIP) series (Figure 2(f)) allows creation of a three-dimensional image, which can be rotated and viewed from 360 degrees.

The MDCT studies are cropped by a trained technologist in our 3D lab and sent to the Hermes workstation. Co-registration can then be performed in a matter of minutes, so the amount of time required is not overly cumbersome. 

Further study will be necessary to determine the benefits of this technique. Questions which will need to be addressed include the following.

- Will this technique improve diagnostic accuracy and decrease interpretation time?
- In which cases will this be required; in all patients or select patients?
- Does this improve evaluation of lucency around metallic devices, suggesting loosening?
- Can this technique be applied to the evaluation of other neurosurgical and orthopedic instrumentation and/or tumors of the musculoskeletal system?

## 5. Conclusion

Accurate assessment of the surgical construct over time is imperative. The co-registration of sequential MDCT studies is technically feasible and may assist in evaluation of the surgical construct. Further study and refinement of this technique will be necessary before clinical implementation. 

## Abbreviations

PET: Positron emission tomography
CT: Computed tomography
MRI: Magnetic resonance imaging
MDCT: Multidetector computed tomography.
